# Observations on the Condition of Asphyxia, or Insensibility, Induced by the Inhalation of Ether; and of the Indications to Be Fulfilled in the Treatment of the Effects of Its Maladministration

**Published:** 1847-06

**Authors:** Charles Searle

**Affiliations:** Bath


*Observations on the condition of Asphyxia, or Insensibility, induced by the inhalation of ether; and of the indications to be fulfilled in the Treatment of the Effects of its Maladministration.* By Charles Searle, M. D., M. R. C. S. E., of Bath.—In the Medical Times of the 19th ult. is a report, made to the Académie de Médecine à Paris, by M. Amussat, of his experiments on animals under the influence of the vapour of ether, in which he said:—"that, during the condition of insensibility induced by the ethereal inhalation, the blood in the arteries was found to be of the venous character, and resumed its florid appearance when the state of unconsciousness had ceased. And that, in animals which had died during the inhalation, the blood was found of the same venous colour in the heart, veins, and arteries." If such are the results of M. Amussat's experiments, they establish, beyond doubt, the correctness of my views on the subject, published in the Medical Times, under date of Jan. 5, to the effect that the condition of the patient under the influence of the ethereal vapour is that of asphyxia ; and that the sedative or narcotic effects of ether as ordinarily administered is of the same character. Its stimulant operation when so administered, that is, by the stomach, I must, however, first explain, or the views I entertain of its inhalation will not be rightly apprehended. To make this understood, it is necessary for me first to observe that life, the attributes of which are heat and motion, is the result of the chemical action developed in the animal system by the agency of the oxygen of the air inspired in its combination with the hydro-carbonaceous constituents of the food existing in the blood—caloric and electricity, the actuating stimuli of all vital phenomena, being evolved in the process. And as this process of chemical combination and electrical evolution, we may justly infer, takes place more particularly in the capillary or hair-like vessels, wherein the particles of the blood are brought into the closest approximation, and in which the final changes in its composition in relation, at least, to the conversion of arterial into venous blood takes place, hence the excitement of these vessels and motion of the blood in the capillary vessels, or organic life of animals ; capillary action being the first visible motion to be discerned in the egg in the process of incubation, and the last to be discovered in animals when death takes place. These views I submitted to the profession as long back as 1830, when I had the honour of reading to the Westminster Medical Society a paper on the subject, and have since enlarged upon in a work recently published, entitled "The Philosophy of Life, Health, and Disease."

Now, the transitory stimulating effects of ether, when received into the blood from the stomach, is in virtue of its composition, it being a compound of carbon and hydrogen ; its absolute composition is carbon, hydrogen, and oxygen ; but the latter, it is presumed, exists in combination with its equivalent of hydrogen in the character of water —that it virtually is, as I say, a hydro-carbon. This, then, we may fairly presume, from its highly inflammable nature, must exist in a looser state of combination, or a condition of greater affinity for oxygen, than that of the fatty or other hydro-carbonaceous constituents of the blood, and would, therefore, when received into the general circulation from the stomach by predilection of affinity, enter immediately into combination with the oxygen of the blood, and a more active combustion, and, by consequence, excitement of the capillaries of the general system, would ensue ; and thus the stimulating qualities of ether, in common with other hydro-carbonaceous alcoholic fluids which operate in like manner. And hence Mr. Spalding, the celebrated submarine operator, found that, when he drank alcoholic beverage, he required a greater amount of air being supplied to him during his operations. But the stimulating qualities of ether are of a very transitory nature, these effects being soon followed by its opposite condition of narcotism ; but before I proceed with these, its effects, as ordinarily administered, I must explain that of the insensibility induced by its inhalation.

The offices of respiration, are, the exhalation of carbonic acid gas, and the reception into the blood of its equivalent of oxygen. Now, in the ethereal inhalation, as the air of the lungs presented to the blood for absorption is not only diluted with this vapour, but in admixture also with this hydro-carbonaceous compound, the latter, when imbibed from the air-cells in admixture with the air, from its greater affinity for oxygen than the constituents of the blood, immediately seizes upon the oxygen, and, combining therewith, thus de-oxidates the blood in the lungs, whereby the blood, thence returned to the heart for general circulation, is deprived of its vitalizing qualifications; and hence defective capillary excitement of the heart and brain, and of all the functions, and thus the condition of insensibility and asphyxia induced: limited, of course, by the extent and period of the inhalation; but involving, necessarily, caution that it is not carried to too great an extent, considering that it virtually consists in a species of burking.

We may now comprehend the narcotic and sedative influence of ether which so soon succeeds to its use when received into the blood from the stomach. It having, as I have already explained, by its union with the oxygen of the blood in the general circulation, produced a certain amount of excitement, other portions of the ether existing in the blood, in their circulation through the lungs, enter into combination with the oxygen of the air imbibed, and here de-oxidating the blood at the expense of the general system, produce effects, though less in degree, yet analogous in kind, to its inhalation—narcotism; and thus its sedative influence and reducing power. And thus are all the operations of ether, I am of opinion, very satisfactorily accounted for, and established as such, I think I may venture to say, by the exprериments adduced of M. Amussat.

Reverting to a former observation, I may be permitted to add, that precisely in character with the operation of ether, in producing both excitement and asphyxia, are the effects of alcohol, though modified in description by the difference that exists between them: alcohol being soluble in water, and mixable with the blood; and ether not so, but much more volatile and combustible. Alcohol is accordingly much more permanently stimulant in its effects, and only asphyxiating when received into the system in a concentrated form, or where the quantity in a more diluted state has been so considerable that the blood in its passage through the lungs has become incapacitated for transmitting oxygen for the purposes of the general system; that is to say, the amount of alcohol present in the vital stream de-oxidates the blood in the lungs before it is returned to the heart for general circulation; and hence the more enduring condition of insensibility which succeeds intoxication, the whole vital stream abounding with its polluting influence.

If the explanation afforded be considered the true one, the circumstances prohibiting it use are obviously those of feeble power, as well as apoplectic predisposition, as the effect of all narcotics and reducing agents on the system is to produce venous congestion; and, from reasons afforded in my work "On the Philosophy of Life," but too lengthy in description to be here adduced, and more especially manifested in the liver and brain; hence there was found, on examination, in the case of death recorded from ethereal inhalation, congestive fulness of the blood-vessels of the brain; and, moreover, that bleeding was found useful in the recovery of dogs which had been rendered apparently dead by its use, as so stated in France. But the most obvious and direct indication to be fulfilled, in the case of its maladministration, is the re-excitement of capillary action by the inhalation of oxygen gas; subsidiary to this, or when this is not immediately available, judicious blood-letting is the next object, followed by the excitement of the skin, and respiratory function of the lungs, by the sudden splash of cold water, followed by galvanism and artificial respiration in extreme cases. And, as an internal stimulant, ammonia, alcoholic fluids, or agents of the like character, being interdicted, nitric acid might prove more useful in these cases than ammonia. In India I once drank an entire drachm in a tumbler of water, and with, I thought soon after, decided exhilarating effects. In addition to these means, warm salt-water clysters are clearly indicated; and, although the last to be noticed, I am not sure that it is the least in importance, an emetic may prove a useful ancillary.—*London. Med. Times.*

